# Levels of Phthalates, Bisphenol-A, Nonylphenol, and Microplastics in Fish in the Estuaries of Northern Taiwan and the Impact on Human Health

**DOI:** 10.3390/toxics9100246

**Published:** 2021-10-01

**Authors:** I-Cheng Lu, How-Ran Chao, Wan-Nurdiyana-Wan Mansor, Chun-Wei Peng, Yi-Chyun Hsu, Tai-Yi Yu, Wei-Hsiang Chang, Lung-Ming Fu

**Affiliations:** 1 Emerging Compounds Research Center, Department of Environmental Science and Engineering, College of Engineering, National Pingtung University of Science and Technology, Neipu, Pingtung 91201, Taiwan; ww0975730185@gmail.com; 2 Institute of Food Safety Management, National Pingtung University of Science and Technology, Neipu, Pingtung 1201, Taiwan; 3 Emerging Compounds Research Center, General Research Service Center, National Pingtung University of Science and Technology, Neipu, Pingtung 91201, Taiwan; 4 School of Dentistry, College of Dental Medicine, Kaohsiung Medical University, Kaohsiung City 807, Taiwan; 5 Faculty of Ocean Engineering Technology & Informatics, Universiti Malaysia Terengganu, Kuala Terengganu 21300, Malaysia; nurdiyana@umt.edu.my; 6 Covalent Bond Technical Services, Ltd., Taipei 104051, Taiwan; werner@covalentbond.com.tw; 7 Department of Environmental Engineering, Kun Shan University, Tainan 71003, Taiwan; ychsu22@yahoo.com.tw; 8 Department of Risk Management and Insurance, Ming Chuan University, Taipei 11103, Taiwan; yti@mail.mcu.edu.tw; 9 Research Center of Environmental Trace Toxic Substances, National Cheng Kung University, Tainan 701, Taiwan; whchang@mail.ncku.edu.tw; 10 Department of Engineering Science, National Cheng Kung University, Tainan 701, Taiwan; loudyfu@mail.ncku.edu.tw

**Keywords:** fish, estuary, phthalates, bisphenol-A, nonylphenol, risk assessment

## Abstract

Due to the sparsity in knowledge, we investigated the presence of various estrogenic endocrine-disrupting chemicals (EEDCs), including phthalates (PAEs), bisphenol-A (BPA), and nonylphenol (NP), as well as microplastics (MPs) in samples of the most widely consumed fish collected from different estuaries in northern Taiwan. We then proceeded to determine the likely contribution that this exposure has on the potential for health impacts in humans following consumption of the fish. Six hundred fish caught from five river estuaries (producing 130 pooled samples) were analyzed to determine how different factors (such as the river, benthic, pelagic, and migratory species) influence EEDCs’ contamination and the possible impacts on human health following typical consumption patterns. The predominant EEDCs was diethyl phthalates (DEP), bis (2-ethylhexyl) phthalates (DEHP), and di-iso-nonylphthalate (DINP) in fish, present at 52.9 ± 77.3, 45.3 ± 79.8, and 42.5 ± 79.3 ng/g dry weight (d.w.), respectively. Residual levels of NP, BPA, and MPs in the fish were 17.4 ± 29.1 and 1.50 ± 2.20 ng/g d.w. and 0.185 ± 0.338 mg/g d.w., respectively. EEDCs and MPs levels varied widely among the five river estuaries sampled due, in part, to differences in habitat types and the associated diversity of fish species sampled. For DEP, the Lao-Jie River and pelagic environments produced the most severely contaminated fish species, respectively. DEP residues were also associated with the burden of MPs in the fish. Based on our analysis, we predict no substantial direct human health risk by EEDCs based on typical consumption rates of estuarine fish by the Taiwanese people. However, other sources of EEDC exposure cannot be ignored.

## 1. Introduction

Endocrine-disrupting chemicals (EDCs), including phthalate (PAEs), bisphenol-A (BPA), and nonylphenol (NP), have raised public concerns due to the activation of estrogenic activities in humans. Once known as the great invention of the 20th century, the manufacturing of plastic products keeps increasing, from 1.7 to more than 300 million tons annually in six decades, and is now an environmental concern [1,2]. The globally large demand for plastics is based on their versatile application range, lightweight, moisture resistance, and low-cost price and then resulting inevitably huge amounts of plastic wastes when they are discarded [3,4]. PAEs, including diethyl phthalates (DEPs) and bis (2-ethylhexyl) phthalates (DEHPs), are commonly found in perfumes, facial washes, cosmetics, plastic toys, food packaging, and wall coverings [5,6]. BPA, which is a monomer, is widely used in the production of polycarbonate plastics, health care products, and epoxy resins and is released into the surrounding environment or food and beverages from plastic containers through depolymerization of these products [7,8]. The shorter-chain alkylphenols, including NP and octylphenols, are degraded from the longer-chain of alkylphenol ethoxylates (APEs) as detergents and surfactants in industrial or domestic products by the aquatic biota to be easily leached into the surrounding environment [7]. The disintegration or decomposition of plastic waste generates secondary waste known as microplastics (MPs), whose average size is less than 5 mm and more than 50 μm, from the combination of chemical, physical, biological, and thermal degradation. Owing to the small size and high specific surface area of MPs, estrogenic endocrine-disrupting chemicals (EEDCs), such as PAEs, BPA, and NP, are easily absorbed or exist on/in MPs [9,10]. MPs release or generate potential toxic substances, particularly for their size and MP-bounded chemicals in natural aquatic environments [9]. However, the processes by which MPs, as a vector of environmental contaminants, causing adverse effects are still unknown. MPs, including MP-bounded EEDCs, have raised public concern due to their widespread existence, properties, and induced toxicity to humans and their adverse effects on the environment [11,12].

Bioaccumulation of estrogenic contaminants, including hormones or EEDCs, has induced the prevalence of feminizing effects, high levels of vitellogenin, and the enhancement of ovotestes prevalence in fish [13,14]. The consumption of MPs by fish may result in adverse effects, including gastrointestinal obstruction, internal abrasion, and pseudo-satiation [15,16]. These EEDCs and MPs might accumulate in human bodies via the consumption of fish. The interference from these EEDCs in endocrine glands causes the duplication, competition, or cessation of the function of the human endogenous hormones [17]. Several studies classified the exposure routes of these EDCs or EEDCs into the human body as ingestion, inhalation, and dermal contact, while several toxic effects, including oxidative stress, disruption in metabolism and energy, translocation, disruption in immune function and neurotoxic and neurodegenerative diseases, were caused [17,18,19]. The other findings also showed the exposure routes and mechanism of toxicity through the potential bioaccumulation in human hair, blood, serum, urine, and even in breast milk [19,20,21,22]. Recent epidemiological studies indicated the effects of PAEs towards gestational diabetes and hypertension among pregnant women and preterm and low birth weight for infants [18,19,20,21,22,23]. Stojanoska et al. (2017) revealed that BPA and PAEs were associated with obesity and glucose metabolism disorders [24]. MPs also could cause toxic effects in the human epithelial cells, such as inducement of tissue damage and chronic irritation [25].

Even so, in recent years, many researchers have recognized the marine MPs, which are possibly widespread in the marine environment and potentially damage the marine ecosystem [25,26,27]. The source of marine MPs in terrestrial and freshwater environments was probably transported from plastic waste. EEDCs, such as MPs, PAEs, BPA, and NP, might be found in estuarine samples, including the sediments or surface water [27]. These chemicals, including PAEs, BPA, NP, MPs, are ingested by aquatic organisms into the food chain in the marine system. Levels of BPA, NP, DEP, di-n-butyl phthalate (DBP), and DEHP were 1.3, 10, 34, 7.8, and 64 mg/g wet weight (w.w.), respectively, in the muscles of European flounder (*Platichthys flesus*) collected from the estuaries and coastal waters of The Netherlands [14]. Omar et al. (2019) investigated BPA concentrations in the estuarine fish captured from Klang River estuary and recorded the highest magnitudes of BPA in *Arius thalassinus* at 2.54 mg/g and *Pennahia anea* at 1.80 mg/g [28]. Chen et al. (2021) investigated MPs in the reef and pelagic fish caught from the southeast coast of Taiwan (Hengchun Peninsula) to show 5.6 ± 5.1 pieces/fish in the gastrointestinal tract [19].

Recently, MPs contamination has been reported in abiotic and biotic products, such as meat, fish, and bottled water [29,30]. Despite the global presence of MPs in fish and shellfish [31,32], MP contamination of estuarine species has rarely been investigated [31]. Diet is a major exposure route for MPs to bioaccumulate in human bodies, particularly from seafood. The plastic disintegrates and is decomposed into the small pieces in the marine environment to cause adverse effects not only to the aquatic species but also involving terrestrial organisms, including the final consumer in the food chain, such as humans [33].

The present study was a survey study to determine the levels of PAEs, BPA, NP, and MPs in fish from five main rivers of northern Taiwan. These estuarine fish were mostly consumed by Taiwanese living in northern Taiwan. Our goal was to investigate PAEs, BPA, NP, and MPs in the estuarine fish and to further assess their risk through ingestion of the estuarine fish in Taiwanese.

## 2. Materials and Methods

### 2.1. Sampling Fields and Sample Collection

**Figure 1:**
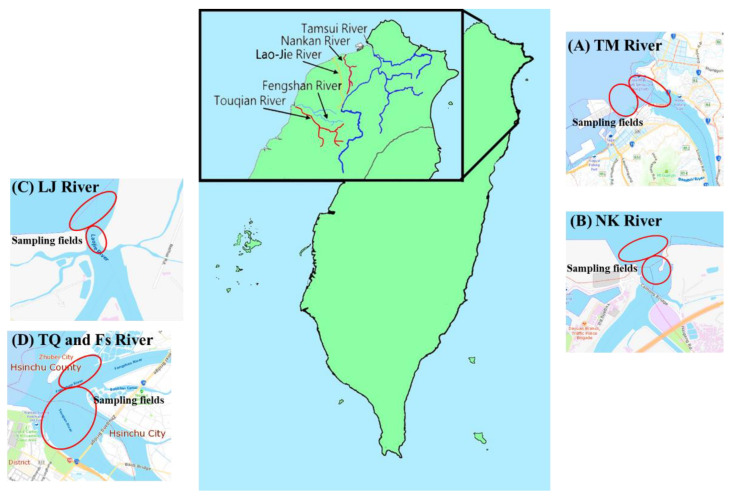
The fish were caught in the estuaries of five main rivers in northern Taiwan. The 130 pooled fish samples were investigated in the present study. The abbreviations of Tamsui, Nankan, Lao-Jie, Fengshan, and Touqian Estuaries are TM, NK, LJ, FS, and TQ, respectively. The sampling locations are shown as red circles in five river estuaries: (**A**) TM, (**B**) NK, (**C**) LJ, and (**D**) FS and TQ.

The sampling areas were located in the estuaries of the five main rivers, including Lao-Jie River (LJ), Nankan River (NK), Touqian River (TQ), Fengshan River (FS), and Tamsui River (TM), in northern Taiwan (Figure 1). The research members used a fish net to catch the fish from a boat between August and December in 2018, and the diverse species are recorded in Table S1. More than 600 fish samples were gathered, and 2–5 fish samples of the same species were pooled together as a pooled fish sample (total number of 130 pooled samples). These 130 pooled samples were divided into 3 groups: benthic, pelagic, and migratory, based on the fish habitat (Table S1). The estuarine fish samples were wrapped in aluminum foil and stored at 0–4 °C in a potable cooler box, and then immediately transferred to the laboratory to be frozen at −20 °C. The parameters of the fish, including fish length and weight, were measured and recorded prior to dissection and grinding. The whole fish soft tissues, including muscle and organs, were separated from the bones by dissection using a stainless-steel apparatus, followed by homogenization in a blender. More than 30 g of homogenate was retained for the determination of MPs before the samples were freeze-dried and then stored at −20 °C prior to chemical analysis.

### 2.2. Extraction, Cleanup, and Analysis of EEDCs

The analytical method of PAEs in this study followed the standard method of the Taiwan Food and Drugs Administration (TFDA) (TFDAA0008.02) with minor modifications. The phthalate standards (>99.0%) were purchased from Supelco/Sigma–Aldrich Co. (St. Louis, MO, USA). The Dibenzyl phthalate standard (a surrogate standard) and isotope internal standards of PAE-d4 (e.g., DEHP-d4) were from Cambridge Isotope Laboratories (Andover, MA, USA). Ethyl acetate, methanol, dichloromethane, acetonitrile, n-hexane, and acetone were obtained from Merck (Darmstadt, Germany). Briefly, 1 g of dried fish powder with 10 μL of labeled internal standard (1 ppm) was mixed with 20 mL methanol/dichloromethane (7:3, *v*/*v*) in a centrifuge tube (50 mL) placed in an ultrasonicator bath to be sonicated for 30 min. The supernatant was collected, and the extraction process was repeated. The extract was concentrated to 2 mL to mix with 10 mL hexane/acetone (4:1, *v*/*v*) prior to passing through a solid phase extraction (SPE) cartridge (HyperSepTM Florisil cartridge, 6 mL, 1000 mg, ThermoFisher SCIENTIFIC, Waltham, MA, USA). The elute was further concentrated to near dryness using a stream of nitrogen that was then redissolved to provide 1 mL eluted solutions of methanol before the analysis. The elute was analyzed using triple-quadrupole liquid chromatography-tandem mass spectrometry (LC/MS-MS) (Acquity UPLC/Waters Xevo TQ-S Micro, Waters, Milford, MA, USA) with a reversed-phase C_18_ HPLC column (Waters XBridge C_18_, 3.5 μm particle size, I.D. × L 2.1 mm × 10 cm). The elute was filtered using a 0.45 μm polytetrafluoroethylene member prior to the injection of 20 μL extract into a sampling loop. Quality assurance and control (QA/QC) followed the methods proposed by TFDA methods, including the solvent and sample blank, limits of detection, recovery rate, and signal-to-noise ratio. The LODs of DBP, DEHP, butyl benzyl phthalate (BBP), diisobutyl phthalate (DIBP), di-iso-nonyl phthalate (DINP), di-n-octyl phthalate (DNOP), dimethyl phthalate (DMP), and DEP were 20, 60, 5, 30, 5, 5, 5, and 5 ng/g d.w., respectively. Most measurements of BBP and DONP were lower than LODs. Each blank test and spike test was conducted on ten samples. The recovery rates of DBP, DEHP, BBP, DIBP, DINP, DNOP, DMP, and DEP were 90.2 ± 9.82, 105 ± 13.5, 79.4 ± 8.54, 78.6 ± 5.62%, 118 ± 14.3, 98.3 ± 5.46, 77.6 ± 5.34, and 103 ± 8.74%, respectively.

Analysis of BPA and NP was modified from a previous report [34]. The native standards of BPA and NP were purchased from Riedel-de Haën Inc. (Seelze, Germany), and the internal isotope standards of ^13^C_12_ NP and ^13^C_12_ BPA were obtained from Cambridge Isotope Laboratories. Four milliliters of ethanol/acetonitrile (1:1, *v*/*v*) was added to 0.5 g of the dried fish powders and sonicated in a centrifuge tube for 10 min and then centrifuged for 10 min to collect the supernatant. The supernatant was concentrated to dryness by gentle nitrogen gas and redissolved in 5 mL n-hexane. A further 2 mL of n-Hexane was added to the remaining bottom layer of acetonitrile in the centrifuge tube with sonication for 10 min, and this process was duplicated, and the supernatants were pooled. The n-hexane solutions extracted from acetonitrile layers were pooled together to be concentrated to dryness to redissolve 2 mL n-hexane further. The Florisil cartridge (SPE) was conditioned by 10 mL n-hexane/acetone (8:2, *v*/*v*) and 10 mL n-hexane. A combination of n-hexane layer solution (7 mL) passed through the SPE cartridge, and then 4 mL of n-hexane was added into the cartridge to gather all the elute. The second fractioned solution of 20 mL n-hexane/acetone (4:1, *v*/*v*) was also passed through the Florisil cartridge, and then the elute was collected. The elute from the fraction of n-hexane and n-hexane/acetone (4:1, *v*/*v*) was pooled to be concentrated to near dryness to be redissolved in 1 mL methanol prior to analysis. The elute was analyzed using an LC/MS-MS (Acquity UPLC/Waters Xevo TQ-S Micro) with a reversed-phase C_18_ UPLC column (Acquity UPLC^®^ BEH C_18_, 1.7 μm particle size, I.D. × L 2.1 mm × 5 cm). QA/QC was followed by TFDA. LODs of BPA and NP were both 2 ng/g d.w., and the recovery rates of BPA and NP were calculated as 75.4 ± 14.9 and 101 ± 2.65 %.

### 2.3. Quantitative Analysis of MPs

The qualitative screening method of MPs in this study was followed by the standard method (NIEA M907.00B) of the Environmental Protection Administration in Taiwan (TEPA) with modification. The MPs ranged in sizes between 5 mm and 50 μm as determined by filter sizes which was our studying target. All of the filters used in this study were weighed after the filters were conditioned, and the experiment was operated in the type II laminar flow bench. The fish samples of 30 g (wet weight) were stored in a 250 mL flask to vigorously mix with 20 mL n-hexane and then to let stand for 1 h to remove the lipid with triplication. The 150 mL of 10% potassium hydroxide (KOH) was poured into the flask and mixed well before being incubated at 60 °C for 24 h. After a vortex of 24 h, the solution waited for 1 h before precipitation. The supernatant was filtered by 5 mm and 50 μm filters, and the remainder was moved to 1 L beaker. After the addition of 500 mL saturated salt solution into the beaker, the solution was stirred for 10 min, and after a 1 h wait, the supernatant was passed through the 5 mm and 50 μm filters. The saturated sodium tungstate (Na_2_WO_4_) solution of 500 mL was added into the beaker and mixed well; then, after 1 h wait, the supernatant was filtered by the 5 mm and 50 μm filters. The filters were collected to be dried and conditioned. The dried plastic particles on the filter were confirmed with a Fourier Transform Infrared Spectrometry (FT-IR), and then the impurity (non-MPs) was removed by a needle under optical microscopy before they were weighed. The weight of the dried MPs was calculated before and after filtration.

### 2.4. Assessment of Human Health via the Estuarine Fish in Northern Taiwan

The health risk in the northern Taiwanese general population was assessed by ingestion of the estuarine fish. The fish caught from these estuaries in northern Taiwan are almost all consumed in the local areas due to these five rivers being located in northern Taiwan (Keelung, Taipei, New Taipei, Taoyuan, and Hsinchu), which is the most densely populated area in Taiwan, contributing approximately 40% of total Taiwanese population and only consisting 14.4% of land in Taiwan.

According to our previous reports with minor modification [35,36], the daily intake (DI) could be represented by the equation of DI_fish_ (ng/kg b.w./day) = (C_estuarine fish_ × AB_absorption rate_ × IR_fish_)/(BW_body weight_), where C_estuarine fish_ is the mean concentration of EEDCs (ng/g d.w.) and MPs (ng/g d.w.) in the estuarine fish. The AB_absorption_ rate was assumed as 100% for the absorption of PAEs, BPA, NP, and MPs in the gastrointestinal (GI) tract in adults. IR_fish_ represents the frequency of fish consumption being the estuarine fish consumption in the general Taiwanese population by different age groups as assumed from the National Food Consumption Database of TFDA [37]. The body weights (BWs) of the general Taiwanese population were as recommended by the Nutrition and Health Survey in Taiwan (NAHSIT) as reported from a survey conducted on the citizens from 2013 to 2016. The probabilistic estimate method of a Monte Carlo simulation (@Risk 5.5 for Excel, Palisade Corporation, NY, USA) was used to evaluate uncertainty analysis. The risks of non-cancer and cancer assessment for EEDCs or MPs via the estuarine fish levels were also evaluated in the present study as follows:

(1) $$\mathrm{Chronic}\;\mathrm{daily}\;\mathrm{intake}\;\left(\mathrm{CDI}\right)=\frac{\mathrm{DIestuary}\;\mathrm{fish}\;\times \mathrm{EF}\;\times \;\mathrm{ED}}{\mathrm{AT}\;\times 365}\;$$

(2) $$\mathrm{Hazard}\;\mathrm{quotient}\;\left(\mathrm{HQ}\right)=\frac{\mathrm{CDI}}{\mathrm{RfD}}\;$$

(3) Cancer risk (R)= CDI × Cancer slope factor(CSF) 

The CDI was calculated by modifying the equation from preview studies. The EF is exposure frequency per year (days/year), which is defined as 365 days/year, and ED is defined as exposure duration (years). AT (average lifespan) in this study was defined as 75.5 and 84 years old for men and women, respectively, according to the Department of Statistics, Ministry of Health and Welfare [38], which investigated the mean AT in Taiwan in 2018 [39]. The oral reference dose (RfD) for DEP, DBP, DEHP, and BPA was set with reference to the US EPA Integrated Risk Information System (IRIS) and was 0.8, 0.1, 0.02, and 0.05 mg/kg/day, respectively [40]. The RfDs of DIBP and DINP were 0.100 and 0.059 mg/kg/day, respectively, as followed from a previous study [41]. RfD of NP referenced from Lee’s report was 0.005 mg/kg/day [42]. The cancer slope factor (CSF) of DEHP is 0.014 for oral exposure [43]. The maximum acceptable oral doses of the toxicants are defined as RfDs, and the estimation of cancer risk correlated with carcinogens is from the CSF of the USEPA. The critical value of non-cancer risk (HQ) is 1.00, indicating that the adverse effects are induced over the critical value of 1.00. A R value exceeding 10^−6^ represents the possible carcinogenic potential of the chemical [43].

### 2.5. Statistical Analysis

Levels of PAEs, BPA, NP, and MPs more than 60% in the surveyed samples which were over LODs were used for further statistical analysis. Measurements of PAEs, BPA, NP, and MPs that were below LODs were treated as 1/2 LODs. The nonparametric methods of Mann–Whitney *U* tests and Kruskal–Wallis *H* tests were used to assess significant differences in fish concentrations of PAEs, BPA, NP, and MPs in the five estuaries and three fish habitats. Spearman’s rank correlation tests were conducted to identify the relationships among EEDCs and MPs in the estuarine fish. Odds ratios of EEDCs and MPs in estuarine fish between river estuaries or fish habitats were examined by the logistic regression models. Levels of PAEs, BPA, and NP in the fish were used to estimate MP values of the estuarine fish by a multiple stepwise linear regression model after logarithmic transformation because these values fulfilled the logarithmically normal distribution. The statistical analyses were performed using SPSS version 12.0.

## 3. Results

Table 1 shows the levels of EEDCs and MPs in fish caught from northern Taiwan estuaries. Six PAEs (DMP, DEP, DBP, DIBP, DEHP, and DINP) were frequently detected in fish from five estuaries and the predominant compounds were DEP (52.9 ± 77.3 ng/g d.w.), DEHP (45.3 ± 33.1 ng/g d.w.), and DINP (42.5 ± 79.3 ng/g d.w.). There were no significant differences in PAE levels among five estuarine fish (LJ: 219 ± 108 ng/g d.w., NK: 179 ± 133 ng/g d.w.; TQ: 210 ± 128 ng/g d.w.; FS: 175 ± 76.3 ng/g d.w.; TM: 213 ± 185 ng/g d.w.). The fish samples from LJ had a higher magnitude of DEP (69.0 ± 54.8 ng/g d.w.) compared with those captured from NK (46.5 ± 101 ng/g d.w., *p* < 0.001), TQ (64.9 ± 81.1 ng/g d.w., *p* = 0.154), FS (26.1 ± 24.9 ng/g d.w., *p* = 0.006), and TM (52.6 ± 79.0 ng/g d.w., *p* = 0.004). DBP levels (39.2 ± 18.7 ng/g d.w.) in TQ estuarine fish were significantly higher than those in fish collected from LJ (17.5 ± 13.2 ng/g d.w., *p* < 0.001), NK (11.3 ± 3.95 ng/g d.w., *p* < 0.001), and TM (17.2 ± 16.1 ng/g d.w., *p* < 0.001), while DBP in FS fish (30.4 ± 31.9 ng/g d.w.) was significantly higher than that in NK fish (*p* = 0.008). For DINP, the significantly higher levels in fish were found in TM (59.9 ± 107 ng/g d.w.), NK (53.1 ± 73.4 ng/g d.w.), and LJ (35.5 ± 58.2 ng/g d.w.) compared with those in TQ (9.11 ± 12.1 ng/g d.w.) and FS (12.2 ± 11.1 ng/g d.w.). DEHP in fish had significantly higher levels in LJ (61.2 ± 50.6 ng/g d.w., *p* = 0.013) than in NK (34.7 ± 14.2 ng/g d.w.), while the level of DEHP in fish in FS (55.6 ± 48.3 ng/g d.w., *p* = 0.056) was borderline-significantly higher than NK. As well, DEHP in TQ (39.3 ± 16.9 ng/g d.w., *p* = 0.383) and TM (42.7 ± 26.0 ng/g d.w., *p* = 0.318) was non significantly higher compared with that in NK. EEDC levels of NP and BPA in fish were not significantly different between LJ (NP and BPA (NP/BPA): 14.8 ± 17.1 and 1.87 ± 2.75 ng/g d.w., respectively), NK (NP/BPA: 13.3 ± 23.9/1.85 ± 3.13), TQ (NP/BPA: 17.1 ± 34.8/1.63 ± 2.50), FS (NP/BPA: 25.2 ± 34.2/<LOD), and TM (NP/BPA: 19.0 ± 33.5/1.24 ± 0.971). Finally, MPs from the estuary fish also showed a comparable weight among five river estuaries including LJ, NK, TQ, FS, and TM with 0.232 ± 0.346, 0.229 ± 0.509, 0.114 ± 0.103, 0.154 ± 0.136, and 0.169 ± 0.299 mg/g d.w., respectively.

**Figure 2:**
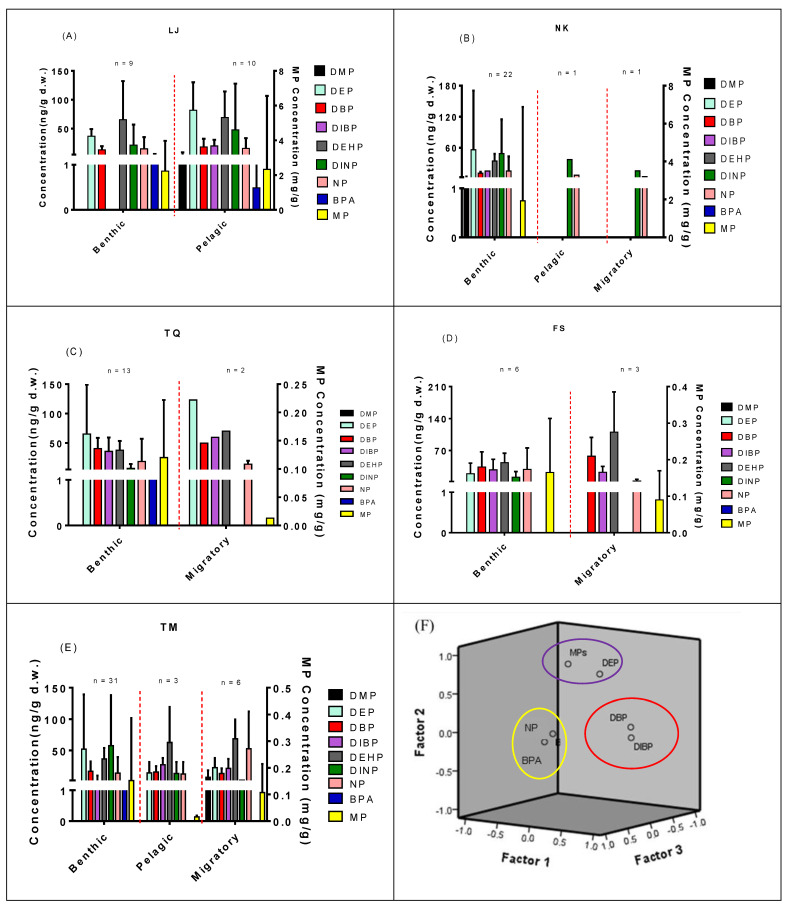
Distribution of EEDCs in estuarine fish samples collected from northern Taiwan. The estuarine fish could be assigned into three groups (benthic, pelagic, and migratory) based on the fish habitat. The estuarine fish levels of PAEs, BPA, NP, and MPs in the estuarine rivers were as follows: (**A**) Lao-Jie (LJ), (**B**) Nankan (NK), (**C**) Touqian (TQ), (**D**) Fengshan (FS), (**E**) Tamsui (TM), and (**F**) Principal component analysis for EEDC residues in the estuarine fish.

According to the fish habitat, benthic, pelagic, and migratory fish were determined (Table 2). Benthic (46.5 and 37.5 ng/g d.w., *p* = 0.133 vs. *p* = 0.003) and pelagic (61.7 and 39.6 ng/g d.w., *p* = 0.041 vs. *p* = 0.067) fish had elevated DEP and DINP levels compared with migratory fish (22.5 and 3.58 ng/g d.w.), but benthic fish (39.0 ng/g d.w.) had the lower DEHP level than pelagic (65.0 ng/g d.w., *p* = 0.012) and migratory fish (72.8 ng/g d.w., *p* = 0.001). The other EEDCs in fish did not show significant differences in DMP, DBP, DIBP, BPA, NP, and MPs among the three fish habitat areas. Concentrations of EEDCs and MPs among three groups of fish habitat in each river estuary are presented in Figure 2A–E). The variation of EEDC levels in estuarine fish was various among the five river estuaries. The variations of DMP, DEP, DBP, DIBP, DEHP, DINP, NP, and BPA levels in fish, were examined by factor analysis, such as the estuary where fish were collected (e.g., Tamsui Estuary), and fish habitat (e.g., benthic) to explain 66.8% of the total variance at the eigenvalue > 1.00 (Figure 2F). For the eigenvalues of DMP, the estuary where fish were collected and fish habitat were lower than 1.00, and the variables were independent among all variables. The variables of DEHP and DINP were excluded due to the difficulty in assigning them into an independent factor group. Three groups (PC1, PC2, and PC3) of EEDCs and MPs in fish were determined by the principal component analysis (PCA) in Figure 2F to show DBP and DIBP in PC1 (explaining 28.5% of total variance; eigenvalue = 1.71), DEP and MPs in PC2 (explaining 20.3% of total variance; eigenvalue = 1.22), and NP and BPA in PC3 (explaining 17.9% of total variance; eigenvalue = 1.075).

Table 3 showed the Spearman’s rho correlation coefficients among EEDCs and MPs in 130 pooled samples of estuarine fish. The DEP level in fish were significantly correlated with DBP (r = 0.199, *p* = 0.023), DINP (r = 0.373, *p* < 0.001), NP (r = 0.261, *p* = 0.003), and MPs (r = 0.243, *p* = 0.005) in fish. DBP was significantly related with DIBP (r = 0.448, *p* < 0.001), DEHP (r = 0.259, *p* = 0.003), and BPA (r = 0.218, *p* = 0.013). DEHP (r = 0.355, *p* < 0.001) and DINP (r = −0.199, *p* = 0.023), respectively, were significantly associated with DIBP as well as NP which was significantly linked into BPA (r = 0.217, *p* = 0.013). The logistic regression model was used to examine the odds ratios (OR) of EEDCs and MPs in fish among the river estuaries or fish habitat. Significant ORs of DEP between the groups of the river estuaries (the location where the fish captured) or fish habitat was only shown among EEDCs and MPs (Table 4). The results suggested that DEP in LJ presented a significantly higher OR of 1.01 (95% confidence interval (CI) between 1.01 and 1.07, *p* = 0.016) than that in FS. Pelagic fish had a significantly higher level of DEP (OR = 1.02, 95% CI = 1.00–1.05, *p* = 0.049) compared with migratory fish. The associations between MPs and EEDCs were examined by multiple stepwise linear regression models. The fish level of logMPs was significantly predicted by logDEP in fish before (crude: B = 0.288, adjusted R = 0.259, *p* = 0.002) and after (adjustment: B = 0.284, adjusted R = 0.235, *p* = 0.003) the river estuaries and fish habitat were adjusted (Table 5).

**Figure 3:**
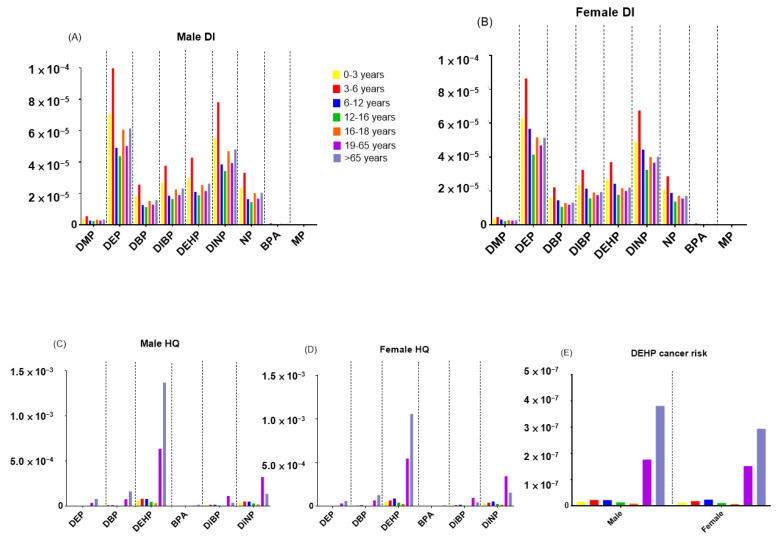
The estimated health risk assessment EEDCs through the consumption of estuarine fish collected from northern rivers, Taiwan, for different populations among age groups:(**A**) DI values of Male (**B**) DI values of Female (**C)** HQ values of Male (**D**) HQ values of Female (**E**) The values of cancer risk for DEHP.

The mean DIs of different age groups were calculated for individual EEDCs through the consumption of estuarine fish and are shown in Figure 3A,B for males and females, respectively. Children, including infants, toddlers, young children, and school-age children, were exposed to higher EEDC DIs than adolescents and adults, based on relatively low BW for children. The highest mean estimated DIs of ΣPAEs, BPA, NP, and MPs were evaluated for 3–6 ages as 29.0 × 10^−5^, 1.13 × 10^−6^, 3.31 × 10^−5^, and 3.56 × 10^−7^, respectively, among the age groups. Figure 3 C, D shows the mean estimated HQs of seven EEDCs, such as DEP, DBP, DEHP, DIBP, DINP BPA, and NP, for males and females in the different age groups, and our values were notably lower than 1.00, indicating no non-cancer risks were found in the present study. The values (Rs) of cancer risk for DEHP ranged between 1.36 × 10^−^^8^ and 1.80 × 10^−^^7^ and between 7.32 × 10^−^^9^ and 1.55 × 10^−^^7^ in males and females, respectively, and were significantly below the critical value of 1.00 × 10^−^^6^. No cancer risk was found in our age groups.

## 4. Discussion

This study aimed to investigate EEDCs, including PAEs, BPA, and NP and MPs, in fish caught from the main five river estuaries in northern Taiwan. According to our investigation in Table 1 and Table 4, PAEs in estuarine fish presented as the most polluting chemicals among EEDCs in fish. PAEs in the estuarine fish captured from LJ showed the most severe contamination compared with those in the other river estuaries. There were no significant differences in levels of BPA, NP, and MPs in fish among these five river estuaries. EEDCs, such as PAEs, BPA, and NP and MPs, in Taiwanese rivers are probably from plastic waste in landfill, domestic wastewater, and riverside plastic factories, and these chemicals enter the ocean through the estuary [15,43]. EEDCs contamination in the estuarine fish might be related to high industrialization and urbanization [14,44]. It is still unknown whether PAEs, BPA, and NP in freshwater and river sediment affect levels of these chemicals in estuarine fish majorly. Estuarine fish survive at the junction of the river and sea with the resultant fluctuation in salinity through all their lives which possibly results in it experiencing a substantial variability in salinity and body burden of EEDCs from the fluctuation of freshwater discharge and ocean current at any time. Estuarine fish exposure to EEDCs might have a minor association with the water level, tidal current, salinity, and other environmental factors.

The classification of fish habitat in this study was not reflected in high and low trophic levels in the estuarine system. EEDCs, including PAEs, BPA, and NP, were not persistent pollutants in fish and their half-life in fish is short compared with persistent organic pollutants, such as dioxins [45,46]. Our results might be consistent with the previous reports focused on the TM water system, indicating that extremely low BPA concentrations were bioaccumulated in the fish from the TM system compared with DEHP and NP [44]. Prof. Lee also revealed that levels of PAEs, BPA, and NP in the river sediment (μg/kg organic carbon) did not have a good correlation with those in river fish [44]. The results suggested that sediment EEDC levels did not directly affect ingestion of these EEDCs by fish, but it might be associated with habitat environment, feeding strategy, metabolic rate in the different species, and freshwater discharge, suspended particles, and octanol-water partition coefficients of EEDCs [44,47,48].

For MPs in estuarine fish, weighed-based MP levels investigated in the present study were different from number-based MP levels in most studies of the aquatic ecosystem. No significant differences in fish MP levels among five river estuaries and fish habitat were found (Table 1, Table 2 and Table 4 and Figure 2). In the distribution of MPs in fish from the previous Taiwanese studies, MP number-based levels in the fish gastrointestinal tract were not significantly different between demersal and benthopelagic species in the river [49] or between the reef and pelagic fish in the coastal water [15]. Tien et al. (2020) showed that MPs in river fish had an obvious positive correlation with suspended particles and negative associations with PH values and conductivity, indicating that the river water quality parameters had significant potential effects on MPs number-based levels [50]. Plastic debris (98%) MPs ingested by marine fish in Taiwan might be associated with human activity but were not related to biotic factors (i.e. biomagnification), the length and weight of fish, trophic level, and taxonomic family of fish [15]. Park et al. (2020) revealed that MPs ingestion by fish was more closely linked to the habitat environment rather than feeding habit. The finding from Park’s study was not consistent with our result, revealing that MP weighed-based levels were not correlated with fish habitat and feeding habits [50]. The Taiwanese study reported the river fish which were collected might like to eat large fragmented MPs from water as well as long fibrous MPs from sediments [49].

Compared with the previous studies reporting EEDC levels in marine fish [51,52,53], not many studies reported levels of PAEs, BPA, and NP in estuary fish [14,39,54,55,56,57,58,59]. Our levels of EEDCs were consistent or inconsistent with the previously described reports, probably due to variation of land-based pollution, diverse fish species, and geographic differences. The materials from the products of PAEs, BPA, and alkylphenols on land are contributing to more than 80% of marine pollution, and the estuary is one of the severely contaminated sources due to wastewater discharge, industrial and domestic release, agricultural runoff, and riverside pollution [60]. EEDC levels in estuarine fish are related to those in water and sediments in the aquatic environment, particularly for the estuarine environment [14,28,51,54].

In Table 3 and Figure 2, the significant associations of MPs and DEP, NP and BPA, and DBP and DIBP were examined by PCA and Spearman’s correlation tests. We found that MPs in the estuarine fish could only be significantly predicted by fish levels of DEP after river estuary and fish habitat were adjusted (Table 5). Plastic waste could be dissected by weathering, mechanical abrasion, and photodegradation in the marine environment, or microplastic beads from cosmetics or sunscreens, to become MPs [51]. MPs contain large quantities of toxic substances not only from plasticizers during the manufacture but also adsorption of PAEs due to their large surface-to-volume ratio [51,61,62].

The mean values of DIs, HQs, and Rs for males and females in the different age groups are shown in Figure 3. The main contributors to the total EEDCs daily intake through estuarine fish were the consumption of DEP and DINP in the present study. Inversely, the mean HQs of NP via the ingestion of estuarine fish for the different age populations, including males and females, had the highest magnitudes among the HQs of EEDCs, probably due to the low value of RfD for NP. Although the age group 6–12 showed obviously higher DIs compared with the other age groups after ingesting estuarine fish, no significant differences in DI values were found between the gender in the same age group. Our results showed that Taiwanese exposure to PAEs, BPA, and NP did not present remarkable health risks based on the estimation of mean DIs, HQs, and Rs in the present study. Compared with our results in the present study, similar findings were also announced in the studies by Lee et al. (2015), Lee et al. (2020), and Wei et al. (2011) to obtain nonsignificant potential effects on human health after assessing non-cancer and cancer risks following the consumption of the marine fish [42,46,63]. Owing to the high fish consumption rates in the ordinary diet of several Asian counties, including Taiwan and Taiwanese, who prefer to consume wild marine fish [42,63,64], the ingestion of contaminated marine fish is one of the important exposure routes in Taiwan. Although the Taiwanese exposure to EEDCs from estuarine fish did not cause remarkable risks to human health, it cannot be concluded that it might not pose potential health effects based on a single-exposure route due to human exposure to EEDCs via multiple pathways. Humans are easily exposed to PAEs, BPA, NP, and MPs mainly due to the widespread existence of PAEs, BPA, NP, and MPs in the environment (e.g., indoor dust or smoking) and foodstuff (e.g., pork or chicken), indicating that these chemicals can enter into human bodies through various pathways. Consumption of estuarine fish is one of the dietary routes for humans. Compared with the risk-assessment studies in Taiwan [65,66,67,68,69], our results, which examined Taiwanese exposure to these chemicals via dietary of the estuarine fish, are presented as the minor contribution. Future studies are encouraged to assess human risks from emerging contaminants, such as brominated fire retardants or pharmaceutical and personal care products, via multiple exposure routes.

## 5. Conclusions

This study was the first report to address the impact of PAEs, BPA, NP, and MPs from estuarine fish after ingestion on human health in Taiwan. Although the estuarine fish were diverse and the distribution of EDDCs in the estuarine fish among five river estuaries and fish habitats were inconsistent, the residues of DEP, DINP, and DEHP in fish were predominant in the present study. No significant differences in weighed-based MPs in the estuarine fish were shown among five river estuaries and the fish habitat. Log MPs could be significantly predicted by the estuarine fish levels of log DEP before and after the river estuary, and fish habitat was adjusted. No remarkable risks to Taiwanese health were found after Taiwanese consumed the estuarine fish based on an assessment of non-cancer and cancer risks.

## Figures and Tables

**Table 1:** Concentrations of PAEs, NP, BPA, and MPs in the estuarine fish from northern Taiwan river estuaries (ng/g d.w.).

	LJ (n = 24) ^a^ Mean ± SD	NK (n = 29) ^a^ Mean ± SD	TQ (n = 16) ^a^ Mean ± SD	FS(n = 14) ^a^ Mean ± SD	TM (n = 47) ^a^ Mean ± SD	Total (n = 130) Mean ± SD	*p*-Value
PAEs	219 ± 108	179 ± 133	210 ± 128	175 ± 76.3	213 ± 185	202 ± 144	0.322
	(73.7–527)	(64.5–592)	(77.7–451)	(80.8–336)	(64.5–980)		
DMP	3.13 ± 3.08	2.75 ± 0.931	<LOD	<LOD	3.08 ± 4.00	2.90 ± 2.80	0.644
	(<LOD-17.6)	(<LOD-6.40)			(<LOD-29.9)		
DEP	69.0 ± 54.8	46.5 ± 101	64.9 ± 81.1	26.1 ± 24.9	52.6 ± 79.0	52.9 ± 77.3	0.003 *
	(11.7–247)	(<LOD-495)	(<LOD-248)	(<LOD-64.3)	(<LOD-426)		
DBP	17.5 ± 13.2	11.3 ± 3.95	39.2 ± 18.7	30.4 ± 31.9	17.2 ± 16.1	20.1 ± 18.7	<0.001 ***
	(<LOD-54.2)	(<LOD-23.3)	(<LOD-83.9)	(<LOD-98.4)	(<LOD-73.5)		
DIBP	17.1 ± 7.12	15.5 ± 2.80	36.0 ± 23.5	22.0 ± 16.2	17.2 ± 7.80	19.7 ± 12.8	<0.001 ***
	(<LOD-42.7)	(<LOD-30.1)	(<LOD-82.8)	(<LOD-71.9)	(<LOD-52.2)		
DEHP	61.2 ± 50.6	34.7 ± 14.2	39.3 ± 16.9	55.6 ± 48.3	42.7 ± 26.0	45.3 ± 33.1	0.108
	(<LOD-229)	(<LOD-79.6)	(<LOD-75.5)	(<LOD-205)	(<LOD-128)		
DINP	35.5 ± 58.2	53.1 ± 73.4	9.11 ± 12.1	12.2 ± 11.1	59.9 ± 107	42.5 ± 79.3	0.070
	(<LOD-254)	(<LOD-286)	(<LOD-46.3)	(<LOD-32.6)	(<LOD-589)		
NP	14.8 ± 17.1	13.3 ± 23.9	17.1 ± 34.8	25.2 ± 34.2	19.0 ± 33.5	17.4 ± 29.1	0.494
	(<LOD-61.1)	(<LOD-117)	(<LOD-137)	(<LOD-123)	(<LOD-135)		
BPA	1.87 ± 2.74	1.85 ± 3.13	1.63 ± 2.50	<LOD	1.24 ± 0.971	1.5 ± 2.2	0.655
	(<LOD-13.5)	(<LOD-17.1)	(<LOD-11.0)		(<LOD-6.16)		
MPs	0.232 ± 0.346	0.229 ± 0.509	0.114 ± 0.103	0.154 ± 0.136	0.169 ± 0.299	0.185 ± 0.338	0.594
(mg/g d.w.)	(<LOD-1)	(0.0041–2.354)	(<LOD)	(<LOD)	(<LOD-2)		

^a^ LJ: Lao-Jie Estuary; NK: Nankan Estuary; TQ: Touqian Estuary; FS: Fengshan Estuary; TM: Tamsui Estuary. * *p*-value < 0.05, *** *p*-value < 0.001.

**Table 2:** Levels of PAEs, NP, BPA, and MPs in the estuarine fish based on the fish habitat or ecological niche (ng/g d.w.).

	Benthic (n = 81) Mean ± SD	Pelagic (n = 14) Mean ± SD	Migratory (n = 12) Mean ± SD	*p-*Value
PAE	182 ± 129	224 ± 116	187 ± 96.4	0.189
	(64.5–733)	(86.4–527)	(80–336)	
DMP	2.59 ± 0.564	3.58 ± 4.04	4.78 ± 7.91	0.445
	(<LOD-6.40)	(<LOD-17.6)	(<LOD-29.9)	
DEP	46.5 ± 76.4	61.7 ± 52.6	22.5 ± 34.8	0.070
	(<LOD-495)	(<LOD-167)	(<LOD-123)	
DBP	19.0 ± 17.7	17.1 ± 12.6	29.0 ± 27.8	0.557
	(<LOD-98.4)	(<LOD-54.2)	(<LOD-85.2)	
DIBP	19.9 ± 14.1	21.2 ± 10.4	23.7 ± 16.4	0.341
	(<LOD-82.8)	(<LOD-42.7)	(<LOD-59.7)	
DEHP	39.0 ± 27.0	65.0 ± 44.9	72.8 ± 50.7	0.001 **
	(<LOD-229)	(<LOD-140)	(<LOD-205)	
DINP	37.5 ± 62.7	39.6 ± 68.5	3.58 ± 3.72	0.014 *
	(<LOD-242)	(<LOD-254)	(<LOD-15.4)	
NP	16.0 ± 29.3	14.1 ± 17.0	29.8 ± 46.9	0.573
	(<LOD-137)	(<LOD-61.1)	(<LOD-135)	
BPA	1.35 ± 1.82	1.28 ± 1.04		0.675
	(<LOD-13.5)	(<LOD-4.89)	<LOD	
MPs	0.163 ± 0.305	2.40 ± 0.366	0.0927 ± 0.135	0.622
(mg/g d.w.)	(0.00342–2.35)	(0.0105–1.39)	(0.00408–0.490)	

* *p*-value < 0.05, ** *p*-value < 0.01.

**Table 3:** The Spearman’s rho correlation coefficients of EEDCs by Spearman’s rank correlation tests (n = 130).

	DEP	DBP	DIBP	DEHP	DINP	NP	BPA
DBP	0.199(0.023) *^a^						
DIBP	0.091(0.302)	0.448(0.000) ***					
DEHP	0.087(0.323)	0.259(0.003) **	0.355(0.000) ***				
DINP	0.373(0.000) ***	0.059(0.507)	−0.199(0.023) *	−0.145(0.101)			
NP	0.261(0.003) **	0.095(0.280)	0.012(0.892)	0.085(0.334)	0.038(0.666)		
BPA	0.150(0.088)	0.218(0.013) *	0.042(0.633)	0.019(0.826)	0.311(0.000) ***	0.217(0.013) *	
MPs	0.243(0.005) **	0.029(0.740)	0.092(0.300)	0.022(0.802)	−0.033(0.708)	0.054(0.539)	0.013(0.887)

^a^ Spearman’s rho correlation coefficient (*p*-value). * *p*-value < 0.05, ** *p*-value < 0.01, *** *p*-value < 0.001.

**Table 4:** Odds ratios (OR) and 95% confidence intervals (CI) of DEP in fish among river estuary and fish habitat.

DEP	River Estuary				
	FS	LJ	NK	TQ	TM
Mean ± SD ^a^	26.1 ± 24.9	69.0 ± 54.8	46.5 ± 101	64.9 ± 81.1	52.6 ± 79.0
OR	Reference	1.01	1.00	1.01	1.01
95% CI of OR	—	1.01–1.07	0.99–1.02	0.99–1.03	0.99–1.03
*p*-value	—	0.016 *	0.483	0.123	0.241
DEP	Fish Habitat				
	Migratory		Benthic	Pelagic	
Mean ± SD ^a^	23.2 ± 34.4		51.7 ± 87.0	61.6 ± 52.6	
OR	Reference		1.01	1.02	
95% CI of OR	—		0.99–1.03	1.00–1.05	
*p*-value	—		0.285	0.049 *	

^a^ Unit: ng/g d.w. * *p*-value < 0.05.

**Table 5:** The chemicals of EEDCs were used to predict MPs by simple stepwise linear regression models.

Dependence	Independence			
	EDCs	B	Adjusted R	*p*-Value
Log MPs				
Crude	LogDEP	0.288	0.259	0.002 **
Adjustment ^a^	LOgDEP	0.284	0.235	0.003 **

^a^ River estuary and fish habitat were as the confounders. ** *p*-value < 0.01.

## Data Availability

The data presented in this study are available on request from the corresponding author.

## Supplementary Materials

The following are available online at https://www.mdpi.com/article/10.3390/toxics9100246/s1; Table S1 Estuarine fish samples classified as benthic, pelagic, and migratory fish. Table S2. Eigenvalues and explained variances of the rotated principal components (RPCs) of EEDCs in the estuarine fish. Table S3. Compilation of assessments of human health based on estimates of consumption of estuarine fishes contaminated from the northern rivers of Taiwan in male populations. Table S4. Compilation of assessments of human health based on estimates of consumption of estuarine fishes contaminated from the northern rivers of Taiwan in female populations.
