# Supplementary material for: Levels of Phthalates, Bisphenol-A, Nonylphenol, and Microplastics in Fish in the Estuaries of Northern Taiwan and the Impact on Human Health
Source: Toxics. 2021 Oct 1;9(10):246. doi: 10.3390/toxics9100246 (PMC8540681; doi:10.3390/toxics9100246)
Supplement: Supplementary file 1 [file toxics-09-00246-s001.zip › toxics-1343585-supplementary.pdf]

toxics

Supplementary Materials: Levels of Phthalates, Bisphenol-A, Nonylphenol, and Microplastics in Fish in the Estuaries of Northern Taiwan and the Impact on Human Health

I-Cheng Lu, How-Ran Chao, Wan-Nurdiyana-Wan Mansor, Chun-Wei Peng, Yi-Chyun Hsu, Tai-Yi Yu, Wei-Hsiang Chang and Lung-Ming Fu

Table S1. Estuarine fish samples are classified as benthic, pelagic, and migratory fish.

Habits	scientific name
Benthic	1. *Atule mate*
	2. *Argyrosomus japonicas*
	3. *Arius maculatus*
	4. *Bothus mancus*
	5. *Calappidae*
	6. *Chelon macrolepis*
	7. *Chrysochir aureus*
	8. *Dasyatidae*
	9. *Gerres decacanthus*
	10. *Lagocephalus gloveri*
	11. *Leiognathus equulus*
	12. *Miichthys miiuy*
	13. *Pennahia microcephalus*
	14. *Portunidae*
	15. *Portunus trituberculatus*
	16. *Scomberoides lysan*
	17. *Secutor indicius*
	18. *Sillago asiatica*
	19. *Synodus fuscus*
	20. *Terapon jarbua*
Pelagic	21. *Atropus Atropos*
	22. *Nibea albiflora*
	23. *Pampus argenteus*
	24. *Prionace glauca*
Migratory	25. *Megalaspis cordyla*
	26. *Moolgarda cunnesius*
	27. *Mugil cephalus*
	28. *Nematelosa nasus*
	29. *Parapristipoma trilineatum*

Table S2. Eigenvalues and explained variances of the rotated principal components (RPCs) of EEDCs in the estuarine fish.

component	Initial Eigenvalues			Extraction Sums of the Squared Loadings			Rotation Sums of the Squared Loadings
	Total	% of Variance	Cumulative %	Total	% of Variance	Cumulative %	Total
1	1.710	28.507	28.507	1.710	28.507	28.507	1.582
2	1.217	20.279	48.786	1.217	48.786	48.786	1.250

3	1.075	17.917	66.703	1.075	66.703	66.703	1.171

Rotated Component Matrix

	Component		
	1	2	3
DBP	.868	.122	.088
DIBP	.844	-.024	.045
DEP	.287	.732	-.049
MPs	-.147	.832	.038
NP	-.002	-.059	.772
BPA	.113	.055	.750

Table S3. Compilation of assessments of human health based on estimates of consumption of estuarine fishes contaminated from the northern rivers of Taiwan in male populations.

Male age		DEHP	DEP	DBP	DIBP	DINP	BPA	NP	DMP	MP
0–3	DI	3.03×10^{-5}	7.06×10^{-5}	1.81×10^{-5}	2.67×10^{-5}	5.57×10^{-5}	7.98×10^{-7}	2.35×10^{-5}	3.93×10^{-6}	2.52×10^{-7}
	HQ	5.83×10^{-5}	3.42×10^{-6}	7.04×10^{-6}	1.03×10^{-5}	3.63×10^{-5}	6.19×10^{-7}	1.84×10^{-4}	—	—
	R	1.63×10^{-8}	—	—	—	—	—	—	—	—
3–6	DI	4.27×10^{-5}	9.98×10^{-5}	2.55×10^{-5}	3.75×10^{-5}	$7.88\times10^{-}$	1.13×10^{-6}	3.31×10^{-5}	5.54×10^{-6}	3.56×10^{-7}
	HQ	8.22×10^{-5}	4.83×10^{-6}	9.93×10^{-6}	1.45×10^{-5}	5.13×10^{-5}	8.73×10^{-7}	2.59×10^{-4}	—	—
	R	2.30×10^{-8}	—	—	—	—	—	—	—	—
6–12	DI	2.10×10^{-5}	4.91×10^{-5}	1.26×10^{-5}	1.85×10^{-5}	3.88×10^{-5}	5.54×10^{-7}	1.63×10^{-5}	2.73×10^{-6}	1.75×10^{-7}
	HQ	8.09×10^{-5}	4.75×10^{-6}	9.78×10^{-6}	1.43×10^{-5}	5.05×10^{-5}	8.59×10^{-7}	2.55×10^{-4}	—	—
	R	2.27×10^{-8}	—	—	—	—	—	—	—	—
12–16	DI	1.88×10^{-5}	4.39×10^{-5}	1.12×10^{-5}	1.65×10^{-5}	3.48×10^{-5}	4.94×10^{-7}	1.45×10^{-5}	2.43×10^{-6}	1.56×10^{-7}
	HQ	4.81×10^{-5}	2.83×10^{-6}	5.82×10^{-6}	8.48×10^{-6}	3.00×10^{-5}	5.11×10^{-7}	1.52×10^{-4}	—	—
	R	1.35×10^{-8}		—	—	—	—	—	—	—
16–18	DI	2.56×10^{-5}	2.89×10^{-5}	1.53×10^{-5}	2.25×10^{-5}	4.75×10^{-5}	6.74×10^{-7}	1.98×10^{-5}	3.32×10^{-6}	2.13×10^{-7}
	HQ	3.28×10^{-5}	9.29×10^{-7}	3.97×10^{-6}	5.78×10^{-6}	2.05×10^{-5}	3.49×10^{-7}	1.04×10^{-4}	—	—
	R	9.19×10^{-9}	—	—	—	—	—	—	—	—
19–65	DI	2.15×10^{-5}	5.04×10^{-5}	1.29×10^{-5}	1.89×10^{-5}	4.00×10^{-5}	5.62×10^{-7}	1.67×10^{-5}	2.79×10^{-6}	1.79×10^{-7}
	HQ	6.35×10^{-4}	3.73×10^{-5}	7.68×10^{-5}	1.12×10^{-4}	3.96×10^{-4}	6.74×10^{-6}	2.00×10^{-3}	—	—
	R	1.78×10^{-7}	—	—	—	—	—	—	—	—
>65	DI	2.63×10^{-5}	6.12×10^{-5}	1.57×10^{-5}	2.31×10^{-5}	4.88×10^{-5}	6.98×10^{-7}	2.04×10^{-5}	3.40×10^{-6}	2.19×10^{-7}
	HQ	2.19×10^{-4}	1.29×10^{-5}	2.65×10^{-5}	3.86×10^{-5}	1.37×10^{-4}	2.33×10^{-6}	6.92×10^{-4}	—	—
	R	6.14×10^{-8}	—	—	—	—	—	—	—	—

Table S4. Compilation of assessments of human health based on estimates of consumption of estuarine fishes contaminated from the northern rivers of Taiwan in female populations.

Ages		DEHP	DEP	DBP	DIBP	DINP	BPA	NP	DMP	MP
0–3	DI	2.71×10^{-5}	6.23×10^{-5}	1.60×10^{-5}	2.35×10^{-5}	4.97×10^{-5}	7.11×10^{-7}	2.07×10^{-5}	3.46×10^{-6}	2.23×10^{-7}
	HQ	4.69×10^{-5}	2.79×10^{-6}	5.75×10^{-6}	8.38×10^{-6}	2.97×10^{-5}	5.05×10^{-7}	1.50×10^{-4}	—	—
	R	1.31×10^{-8}	—	—	—	—	—	—	—	—
3–6	DI	3.75×10^{-5}	8.60×10^{-5}	2.21×10^{-5}	3.24×10^{-5}	6.85×10^{-5}	9.65×10^{-7}	2.86×10^{-5}	4.78×10^{-6}	3.08×10^{-7}
	HQ	6.47×10^{-5}	3.85×10^{-6}	7.93×10^{-6}	1.16×10^{-5}	4.09×10^{-5}	6.97×10^{-7}	2.07×10^{-4}	—	—
	R	1.81×10^{-8}	—	—	—	—	—	—	—	—
6–12	DI	2.44×10^{-5}	5.68×10^{-5}	1.46×10^{-5}	2.13×10^{-5}	4.50×10^{-5}	6.35×10^{-7}	1.88×10^{-5}	3.14×10^{-6}	2.02×10^{-7}
	HQ	8.50×10^{-5}	5.07×10^{-6}	1.04×10^{-5}	1.52×10^{-5}	5.38×10^{-5}	9.16×10^{-7}	2.72×10^{-4}	—	—
	R	2.38×10^{-8}	—	—	—	—	—	—	—	—
12–16	DI	1.80×10^{-5}	4.16×10^{-5}	1.07×10^{-5}	1.56×10^{-5}	3.30×10^{-5}	4.64×10^{-7}	1.38×10^{-5}	2.30×10^{-6}	1.48×10^{-7}
	HQ	4.15×10^{-5}	2.47×10^{-6}	5.09×10^{-6}	7.42×10^{-6}	2.63×10^{-5}	4.47×10^{-7}	1.33×10^{-4}	—	—
	R	1.16×10^{-8}	—	—	—	—	—	—	—	—
16–18	DI	2.18×10^{-5}	2.60×10^{-5}	1.31×10^{-5}	1.91×10^{-5}	4.03×10^{-5}	5.70×10^{-7}	1.69×10^{-5}	2.83×10^{-6}	1.82×10^{-7}
	HQ	2.55×10^{-5}	7.74×10^{-7}	3.12×10^{-6}	4.55×10^{-6}	1.61×10^{-5}	2.74×10^{-7}	8.16×10^{-5}	—	—
	R	7.13×10^{-9}	—	—	—	—	—	—	—	—
19–65	DI	2.02×10^{-5}	4.69×10^{-5}	1.20×10^{-5}	1.76×10^{-5}	3.71×10^{-5}	5.28×10^{-7}	1.56×10^{-5}	2.62×10^{-6}	1.67×10^{-7}
	HQ	5.39×10^{-4}	3.21×10^{-5}	6.61×10^{-5}	9.64×10^{-5}	3.41×10^{-4}	5.81×10^{-6}	1.73×10^{-3}	—	—
	R	1.51×10^{-7}	—	—	—	—	—	—	—	—
>65	DI	2.26×10^{-5}	5.14×10^{-5}	1.32×10^{-5}	1.93×10^{-5}	4.06×10^{-5}	5.79×10^{-7}	1.71×10^{-5}	2.88×10^{-6}	1.83×10^{-7}
	HQ	2.44×10^{-4}	1.45×10^{-5}	2.99×10^{-5}	4.36×10^{-5}	1.54×10^{-4}	2.63×10^{-6}	7.82×10^{-4}	-	-
	R	6.83×10^{-8}	—	—	—	—	—	—	—	—
